# Enhancing urban transformative capacity through children’s participation in planning

**DOI:** 10.1007/s13280-019-01146-5

**Published:** 2019-02-15

**Authors:** Maria Nordström, Mark Wales

**Affiliations:** Department of Work Science, Business Economics and Environmental Psychology, Swedish University of Agricultural Sciences, Alnarp, Sweden

**Keywords:** Children’s participation, Environmental psychology, Sustainability, Sweden, Urban planning

## Abstract

This paper presents a perspective we find lacking in the general discussion on urban transformative capacity, as well as the discourse on children’s environmental experiences and their participation in urban planning processes. Children contribute to the building of urban transformative capacity in several ways. Firstly, they *contribute with new perspectives on the environment* that broaden existing perspectives on human–environment relations in urban planning. Secondly, children’s participation in planning processes *challenges existing power relations* through the creation of new adult–child relationships which in turn transform relations between adults and other actors and agencies. Thirdly, their participation requires changes in *planning practices* and the establishment of new routines in order to plan cities which meet the needs of children.

## Introduction

The rate of urbanisation today is so rapid that we are not able to keep pace in understanding its consequences (Ernstson et al. 2010). On top of this, Ernstson and colleagues contend that the current planning model is not equipped to cope with the increasing uncertainties we are facing. They argue, therefore, that transitions are needed ‘in urban planning and governance, which enable cities to navigate change, build capacity to withstand shocks, and locate sources of experimentation and innovation in face of uncertainty’ (ibid.). In relation to this, the notion of urban transformative capacity has grown out of the need to understand the specific traits of urban settings required to carry out these transitions. With regard to social–ecological systems, transformability refers to ‘the capacity to create untried beginnings from which to evolve a new way of living when existing ecological, economic or social structures become untenable’ (Walker et al. 2004, p. 4). In his conceptualisation of urban transformative capacity, Wolfram (2016) emphasises that it is a measurement of the ability of urban actors, institutions and artefacts to both plan for and carry out such transitions within and across the various systems which comprise our cities. It is, however, vital the differences between sustainable urban transformation and sustainable urban development are understood. McCormick et al. (2013, p. 4) highlight that whilst sustainable urban development focuses primarily on the development of urban areas, sustainable urban transformation ‘places a stronger emphasis on structural transformation processes’ and single out planning as a vital element in such processes. Transformability does not happen by itself—nor urban planning—but is a result of the actions of various agents in and between different networks and institutions. To understand and enhance transformability, we therefore need to increase our awareness of the complex processes influencing our cities, as well as the degree to which different agents and agencies are involved.

One of the merits of the concept of transformability is its stress on the importance of making clear who “transforms”. In other words, who are the agents and agencies shaping and forming our cities today with consequences for the future? This is in accordance with Ernstson et al. (2010, p. 538) who argue that in human-dominated systems ‘the answer to increased resilience might not lie in its ecological dimension, but rather in the social’. In contemporary urban planning, numerous agents actively strive to assert their influence over the processes shaping the development and function of our cities. The process of change in our cities could therefore be said to be determined by the interactions between these agents. This is in line with the adoption of social–ecological approaches found in research on resilience and sustainability that views social and ecological systems as interdependent and cities as constituted of social–ecological processes (Walker et al. 2004; McCormick et al. 2013; Caradonna 2018).

If transformative capacity can be said to represent the ‘power to change’ that emanates from ‘(empowered) actors’ (Wolfram 2016, p. 126), then strategies aimed at managing the balance of power amongst actors are essential to promote the building of transformative capacity. Wolfram goes on to suggest the need to look at ‘how actors and their structured interactions can achieve collective empowerment for systemic transformation’ (ibid.). In support of this, McCormick et al. (2013, p. 5) write that ‘such efforts should be interconnected across sectors and be adapted for specific urban and national policy conditions to ensure empowerment, engagement and collaboration of relevant stakeholders’. Nevertheless, in order to exert influence over the complex array of systems, processes and structures, one must first identify them and find approaches which incorporate the diverse urban stakeholders or actors. This is a matter of giving clarity to the process of urban planning (cf. Castán Broto et al. 2018; Wolfram 2018).

## Background

### Sustainable development and children’s perspectives

Transformability is a core concept in the discourse on sustainability, where the duality of present and future is a recurring theme. With this comes the complexities that arise when ‘acting in the present on the basis of the future’ (Anderson 2010, p. 778). In the same way, this duality is also present in conceptions of children as “becomings” as opposed to “beings” (Uprichard 2008) and is evident in the ‘generalising tendency to “futurity”’ in discourse surrounding children’s relationship with sustainability’ (Horton et al. 2013, p. 250). Until recently, children have been seen as little more than future receivers of sustainable development as opposed to active participants in its achievement in the present. Heft and Chawla (2006, p. 199), however, point out that if sustainable practices are to traverse time, ‘then children must be the bridge conveying their value and ways’.

Similarities have been drawn between the qualities of child-friendly cities and sustainable cities (Björklid 2010; van Vliet et al. 2017) and the UN Convention on the Rights of the Child (UNICEF 1989) suggests that children’s well-being and quality of life should be seen as the ultimate gauge of sustainable development (UNICEF 1996). Central to this notion is meeting children’s needs. Likewise, the identification and fulfilment of social needs has been highlighted as vital in transformative capacity development and sustainability (Wolfram 2016). However, there is a growing body of research pointing out the failure of urban environments to meet children’s basic need for healthy living environments. This includes studies highlighting the toxic effect of traffic exposure during childhood on adolescent lung capacity (Schultz et al. 2015) and the equally toxic effect of noise on children’s capacity to learn and retain memories (Stansfeld et al. 2005). Through children’s sensitivity the quality of the urban environment is reflected. This is a viewpoint stressed by Malone (2015, p. 18), who has suggested that ‘the well-being of children and their participation could serve as both a *maker* and a *marker* of the progress of city or country to meet the challenge of sustainable development’. Children relate to and use their local environment in intense, sense-oriented and physical ways (Björklid and Nordström 2012), making their activity patterns typically different to those of adults (Matthews and Limb 1999; Davison and Lawson 2006). Due to their limited independent mobility, children are dependent on their local environments to meet their needs for healthy development in ways adults generally are not. For this reason, their perspectives on them are also different to adults. They are place specific and detailed, making them ‘important to consider in their own right’ (Christensen et al. 2015, p. 589). In a previous study (Nordström 2010), children aged 12 years old were asked to provide their perspectives on the particularities of the urban contexts in which they live and they were able to both address them and articulate them clearly in written form. Similarly, in a study on children’s perspectives on green spaces and their management, children aged 10–11 years old displayed a clear understanding of the threat plans for densification in their village posed for their social lives and freedom to move around in their neighbourhood (Jansson et al. 2016). Whilst a lack of knowledge on how to include children’s perspectives in the planning process has been found to provide barriers to their inclusion in urban planning (Cele and van der Burgt 2015), Horelli (2007) shows how such particularities can be transformed into the terminology of urban planning and management. However, for this transformation to occur, a process is needed, and this demands a commitment to include and communicate with children in urban planning and management processes.

### Children’s natural capacity as agents of change

Children’s defining activity, unique to childhood, is play. In play, children show that they possess an active disposition towards their physical and social environments. This active disposition has a developmental value for children as they learn about the world around them—exploring it and adapting to it as well as influencing it—in the process of trying out their own capacities and competencies (Seiler 1998) and shaping it to meet their needs (Chawla 2015). As a consequence of the individual’s acceptance of and interest in change as a natural phenomenon during childhood, Pulkkinen (2017) points out that as an adult, he or she will be more likely to be engaged in the future of society and in matters of general concern such as sustainability. Children can therefore be said to have a natural capacity to observe, contribute to and examine the results of change—rendering them agents of change. This is in agreement with Westley et al.’s (2013, p. 12) call for more work from different disciplines, which investigates the ‘role of agency in SES transformation, and our understanding of the vital impact that individuals can have in these processes’. What society can learn from and nurture in children in this respect is the way in which they interact with and learn about the environment, as well as their ability to affect it. Mårtensson (2004, p. 111), in her study of outdoor play in preschools in Sweden, explains how with certain natural elements, such as snow, it can sometimes look as if the children are trying to ‘approach it with their whole body’ and how their concrete way of interacting with their surroundings makes children particularly perceptive to and prepared for change. As a result, children develop a flexible and accommodating relationship with their surroundings which causes the physical environment to take on ‘the role of co-creator in the process of play’ (ibid., p. 112). In other words, play is the result of a child–environment collaboration. Research has shown that even young children’s views can be taken into regard and ‘if barriers to participation are lowered and appropriate assistance is provided, children can share significant views from an early age, even if they cannot express them verbally’ (Derr et al. 2018, p. 33).

### Challenging established planning procedures by involving children

The conceptualisation of the role of children in planning systems is gradually changing, shifting from a view of them as passive beneficiaries to agents of change (Malone and Hartung 2010). Supporting this, there is a substantial body of research recognising children’s participation in urban planning as a key element of sustainable development. Children’s ability to partake in participation builds on opportunities for their informal participation in everyday life and interaction with the environment (Malone 2013; Derr et al. 2018). It is the combination of informal and formal channels of participation which provide the foundation for their agency in processes of change (Hart 1997; Heft and Chawla 2006). It has been argued, however, that for this to occur there need to be ‘structural changes in society as a whole’ (Percy-Smith and Thomas 2009, p. 357) and that children and their participation have a vital role in evening out the balance of power between children and adults (Mason and Bolzan 2009). Similarly, even if they cannot express them verbally Folke et al. (2010) point out that transformational change in social–ecological systems often involves shifts in ‘social network configurations, patterns of interactions among actors including leadership and political and power relations’. In addition, proponents of Transformative Social Innovation (TSI) theory state that ‘social innovations become transformative when they challenge, alter, replace or produce alternatives to well established social relations, and ways of doing things’ (Dumitru et al. 2017, p. 2).

### Including children’s perspectives in urban planning: An example from Sweden

Folke et al. (2010, p. 5) suggest that shocks or perceived crises in relation to social–ecological systems provide opportunities for the re-evaluation of the current system, which in turn may lead to ‘new kinds of adaptability or possibly to transformational change’. We argue that the failure of the current planning model in Sweden to meet the needs of children growing up in cities is such a crisis. Rapid urbanisation and densification combined with the growing number of children in major cities has seen the incapacities of the present system manifest themselves in a number of ways. Research reveals how children’s independent mobility has declined in recent years (Björklid and Gummesson 2013) and spaces for outdoor play are also diminishing (Kylin and Bodelius 2015) with consequences for their healthy development and well-being.

The Swedish urban planning system is now at a tipping point concerning the recognition of children’s environmental interests in urban planning and the role of children in planning. From the mid-1960s and until the mid-1980s, children’s environmental interests were guaranteed by so-called normative planning, i.e. legislation made it compulsory for urban planners to follow norms concerning school grounds, play areas and other outdoor physical urban areas for children including safe walking to school. Traffic planning in residential areas created safe local environments for children (Mårtenson and Nordström 2017). However, since the mid-1980s, it has been the responsibility of each individual municipality to decide how to safeguard children’s outdoor environments. As a result, the position of children’s interests in urban planning has become more complicated, meaning that children’s interests are dealt with differently. An example of this are child impact assessments (CIA), a new planning instrument used to evaluate the impact planning practices might have on children (Nordström 2017). The background to CIAs and other similar attempts is both the previous child-friendly normative urban planning as well as the strong political dedication in Sweden to the UN Convention on the Rights of the Child. Whilst there are no longer any guarantees for outdoor spaces for children, through CIA children can become more involved in the urban planning process than under previous legislation. Thus, when children are invited and allowed to participate in the planning process their role changes from recipient to participant. This change of roles might have consequences for the urban planning system by eventually affording agency to children.

Through the initiative of three governmental institutions, the Swedish Transport Administration (Trafikverket), the Swedish National Board of Housing, Building and Planning (Boverket) and the Swedish National Institute of Public Health (Folkhälsoinstitut), a project was created in 2010 called “It becomes important when it’s for real!” The project was established to support communities in engaging in child-friendly urban planning and provide inspiration for other municipalities and relevant actors to ‘strengthen children’s and young people’s participation and influence in physical planning’ (Trafikverket 2013). The project involved over 1000 children and built on Article 12 of the Convention on the Rights of the Child which acknowledges that children ‘have the right to participate in decision-making processes that may be relevant in their lives and to influence decisions taken in their regard’ (UNICEF 2007, p. 1). The article also recognises ‘the potential of children to enrich decision-making processes, to share perspectives and to participate as citizens and actors of change’ (UNICEF 2007, p. 1). The example we draw on is from the first phase of the project, which ran from 2010 to 2012. This first phase functioned as a starting point for the sharing of experiences, as well as new attitudes, between different municipalities. We see it as an important point of departure for new perspectives on children in urban planning, emphasising the significance of actively involving children in the process and through this their experiences and knowledge.

In Borlänge, a medium sized municipality of 50 000 inhabitants, local schools were involved in the project. The municipality highlighted the need to develop a ‘toolbox’ of different methods for facilitating communication with children of different ages in ‘different types of processes and at different levels of planning’ with the ultimate aim to incorporate them into the daily routines of planning practices (Trafikverket 2013). Furthermore, the project also aimed to increase the interest of schools for planning issues affecting young people with the hopes of creative mutually beneficial partnerships between schools and planning offices. A range of different methods were utilised to investigate a range of issues including safety, the redevelopment of a square and outdoor activities. These methods ranged from study visits, to model building, photography, focus groups and walking tours. Taking into account the different ages of the participating children was crucial in making participation relevant and real. For example, younger children were asked to show their play areas and routes to and from school, whilst teenagers were asked about their social networks and meeting places. In addition, drawing on previous research, methods were chosen for each age group which reflected the varying competencies of the participants. The learning outcome of the project was found to be mutual for planners and children, as well as their intermediaries, such as teachers and parents.

Whilst the Swedish Transport Administration acknowledged the success of the project as a whole and the need to share experiences from the project, the project’s report also underlined the fundamental need to transform the way the contemporary planning system works if children’s perspectives are to be included. The project provides concrete examples of how, through the collaboration of different actors and agencies around the common issue of children’s participation in urban planning, one can both assess and begin to build transformative capacity within the planning system. The title of the project, “It becomes important when it’s for real!”, was a remark made by one of the young people participating in the project. “Reality”, in this sense, goes beyond the realm of the physical and includes the participation and active engagement of children and young people in issues important to them. It also involved providing children with feedback and showing them the results of their participation, such as physical changes made to the built environment. As one participant stated, ‘I am proud that I was able to be involved in affecting everybody’s future!’ (Trafikverket 2013). Children’s desire to become involved in decision-making is vital and the project from Borlänge highlighted the importance of using appropriate methods to investigate real life issues in order to unlock children’s interest and their capacity as agents of change. As Heft and Chawla (2006, p. 212) argue, ‘the very possibility of sustainable development depends on nurturing a generation of children who recognize the connection between human action and environmental sustainability and, most critically, who can imagine themselves as being participants in achieving this end’.

## Discussion

### Essential aspects when transforming the planning process by involving children in the process

Much of previous participatory planning work aimed at incorporating children’s perspectives has been labelled as ‘systems maintaining’ as opposed to ‘systems transforming’ (Heft and Chawla 2006). Percy-Smith and Malone (2001, p. 1) believe transformation is possible, but warn that ‘authentic participation involves inclusion—wherein the system changes to accommodate the participation and values of children—rather than integration—wherein children participate in predefined ways in predefined structures’. Freeman (2006, p. 82) argues that the benefits of ‘a more inclusive system of planning accrue at three points: for children, for society and for the professionals involved’. Similarly, in his argument for urban transformative capacity, Wolfram (2016, p. 127) highlights the need to develop ‘new knowledge of systemic relations between ways of thinking (cultures), organising (structures) and doing (practices)’ in order to understand ‘deficits in meeting social needs’. These viewpoints by Wolfram converge with those by Freeman, Percy-Smith and Malone at the following three essential and interrelated points:

### Recognising the value of children’s perspectives and competencies for society

At the heart of this approach is the acknowledgement of children as urban stakeholders and the need to understand and recognise the value of children’s perspectives and competencies for society. It is about recognising children’s own perspectives, as well as the ‘the multiple realities of children’s lives’ (Freeman and Aikten-Rose 2005, p. 232). Similarly, Malone and Hasluck (2002) argue that we need to look beyond simply changing or designing the physical environment and understand the ‘culture of a community and young people’s role with it’ (107). To aid this process, the development of methods to help us capture, communicate and understand children’s perceptions is crucial (see Cele 2006; Christensen et al. 2015). Ensuring a good participant–method fit ensures children are able to participate on their own terms (Alderson and Morrow 2011) and helps planners come closer to children’s actual experiences of their living environments. This, however, does not exclude the capacities of adults. In their work on developing children’s maps in Geographical Information Systems (GIS) to facilitate children’s participation and influence in urban planning, Nordin and Berglund emphasise the importance of the communicative skills of the people administering the method both in their interactions with the children and their ability to interpret the results for urban planners (Nordin and Berglund 2010). Likewise, the development of new ways of thinking about children’s role in sustainability transitions is equally important if transformation is to occur.

The value of adopting this approach provides planning professionals and the planning system itself with the possibility to ‘build on the understanding generated by those who have the most intimate knowledge [of their local environment]’ (Freeman 2006, p. 83) and will allow them to ‘enhance the fit or congruence between the intentions of the users and their settings’ (Horelli 2007, p. 285) (cf. Gordilho-Souza et al. 2019). As a result, the inclusion of children’s perspectives and competencies in the planning system builds its capacity to transform by recognising children as important urban stakeholders and consequently improving its ability to plan environments in which children can thrive.

### Making clear the position of children in social structures and power relations

This component revolves around the need to both understand and challenge structures of power and societal norms. Matthews (1995), in describing children’s position in society as ‘outsiders’, wrote that ‘the visions of environmental planners and landscape architects implicitly reflect the dominant perceptions of a society, such that groups on the edge become further marginalized by policy making’ (456). Similarly, Cele and van der Burgt (2015, p. 17) highlights a number of the problems, such as ‘unequal economic and political resources’, in managing power imbalances associated with children’s participation in the planning process. In relation to this, Wolfram (2016) highlights ‘community empowerment and autonomy’ as a key component in his conceptual framework of urban transformative capacity and highlights the need to identify ‘conditions of citizen powerlessness and disempowerment’ (127). Central to overcoming such imbalances is the ‘communicative nature of the planning process’ (Horelli 2007, p. 279) which has a key social role to play and key to this is understanding ‘how participation can be organised in such a way that the planning cycle becomes an arena for the learning and capacity-building of young people, as well as for the adults that support them’. (cf. Ziervogel 2019). In order to do this, Freeman and Aitken-Rose (2005, p. 232) believe a ‘new relationship’ needs to be formed that involves planners transforming their role from ‘expert decision-makers acting on behalf of young people, to acting in partnership with the child and youth community’. In support of this, children’s participation has been highlighted as a ‘key strategy in transforming relationships with adults’ (Percy-Smith and Thomas 2009, p. 357). We therefore argue that it is through the acknowledgement of children’s agency and their participation in the planning process that existing social structures and power relations can be identified and challenged in order to contribute to the development of capacity for urban transformation.

### Transforming the planning system by adopting a more versatile understanding of time

The most difficult challenge in achieving sustainable urban transformation lies in implementing new practices and establishing robust routines which satisfy multiple needs and achieve complex aims. We suggest that one way forward might be to adopt a more versatile understanding of time in relation to urban development. All planning activities entail handling change (Lynch 1972). All changes, however, are not of the same character, but take place at different rhythms—some are slower and over a long time, whilst others are instant. They also have very different consequences. Time, and therefore change, is experienced differently by people in different societies, groups and ages. In transforming our cities for the benefit of their citizens, as well as the various psychological, social, economic and ecological systems of which we are part, we should strive to clarify the diverse meanings of change. Children are perhaps our best reminder of this and make us uniquely aware of the fact that their development takes place at different rates during different phases of their development. As a result, they understand, react to and handle events in their lives differently depending on their development needs. Through their inclusion in planning practices we not only remind planners and other involved actors of this fact, but also make children themselves aware of this fact. Their participatory planning experiences can help them learn to direct their attention to the environment in which they live and better understand and accept change. Through this process they also develop their capacity both as children and as future adults to address not only local issues but global issues of importance for the future of their own lives and the societies in which they live.

## Conclusion

This paper began by discussing the critical role of urban planning in the process of building urban transformative capacity. It also acknowledged the links between sustainable development and children’s perspectives and illustrated how children’s way of interacting with and seeing their surroundings provides us with an opportunity to gain new perspectives and knowledge on current urban systems. We then discussed how children can become agents of change through the inclusion of their perspectives in the urban planning process through their participation. Within their role as agents of change, children take on several different roles. Firstly, they contribute with new knowledge and perspectives on the environment that change existing perspectives on human–environment relations in urban planning and enhance the “fit” of urban environments for its users. Secondly, their participation transforms existing power relations through the creation of new adult–child relationships which in turn transform relations between adults and other actors and agencies, as well as human–environment relationships. Thirdly, through their participation children challenge existing routines and ways of thinking about change. Their participation has the potential to act as a catalyst for the transformation of planning practices into new, more sustainable and responsive constellations. Through this process children contribute to the building of the urban transformative capacity of our cities by contributing to the ability of planners to create cities which meet the needs of their youngest residents. This paper has illustrated how children’s issues are increasingly being included in studies on sustainability. Building on this, we therefore call for the inclusion of children’s issues as part of the broader discussion on urban transformative capacity in the hopes that children’s perspectives and experiences can help contribute to new ways of thinking about change.
